# Functional Characterization of Facilitative Glucose Transporter 4 With a Delay Responding to Plasma Glucose Level in Blunt Snout Bream (*Megalobrama amblycephala*)

**DOI:** 10.3389/fphys.2020.582785

**Published:** 2020-10-15

**Authors:** Hualiang Liang, Sahya Maulu, Ke Ji, Xianping Ge, Mingchun Ren, Haifeng Mi

**Affiliations:** ^1^Key Laboratory for Genetic Breeding of Aquatic Animals and Aquaculture Biology, Freshwater Fisheries Research Center, Chinese Academy of Fishery Sciences, Wuxi, China; ^2^Wuxi Fisheries College, Nanjing Agricultural University, Wuxi, China; ^3^Tongwei Co., Ltd., Chengdu, China; ^4^Healthy Aquaculture Key Laboratory of Sichuan Province, Sichuan, China

**Keywords:** blunt snout bream, glucose transporter 4, glycometabolism, delay action, insulin

## Abstract

Facilitative glucose transporter 4 (GLUT4) plays a central role in mediating insulin function to increase glucose uptake in glucose metabolism homeostasis. In this study, the function and localization of GLUT4 in blunt snout bream (*Megalobrama amblycephala*) were first investigated, and then, the response measured as carbohydrate level, was analyzed. The results showed that the cDNA sequence of GLUT4 in blunt snout bream (MaGLUT4, GenBank accession no: MT447093) was 2868 bp in length, and the corresponding mRNA contained a 5′-UTR region of 513 bp and a 3′-UTR region of 837 bp. MaGLUT4 had an open reading frame of 1518 bp and was encoded by 505 amino acids. Its theoretical isoelectric point and molecular weight was 6.41 and 55.47 kDa, respectively. A comparison of these characteristics with BLASTP results from the NCBI database showed that MaGLUT4 had the highest homology with Cypriniformes fish, with MaGLUT4 and GLUT4 of other Cypriniformes clustered in the phylogenetic tree with other GLUT1–4 amino acid sequences. Compared with the results from the *homo_sapiens* and *mus_musculus* data sets, some mutations were observed in the GLUT4 amino acid sequence of these aquatic animals, including an FQQI mutation to FQQL, LL mutation to MM, and TELEY mutation to TELDY. MaGLUT4 was constitutively expressed in the muscle, intestine, and liver, with the highest mRNA level observed in muscle. Furthermore, the predicted tertiary structure and results of immunohistochemical staining showed that MaGLUT4 was a transmembrane protein primarily located in the plasma membrane, where it accounts for 60.9% of the total expressed, according to an analysis of subcellular localization. Blood glucose level peaked within 1 h, and the insulin level peaked at 6 h, while the mRNA and protein levels of GLUT4 showed an upward trend with an increase in feeding time and decreased sharply after 12 h. These results confirmed that MaGLUT4 was mainly distributed in muscles and crosses the cell membrane. The changes in the insulin, mRNA, and protein levels of MaGUT4 lagged far behind changes in blood glucose levels. This delay in insulin level changes and GLUT4 activation might be the important reasons for glucose intolerance of this fish species.

## Introduction

In aquatic animals, carbohydrates are widely used in feed as energy sources. On the one hand, carbohydrates are more readily available and much less expensive than protein and can substitute for protein to prevent the catabolism of expensive protein nutrients to meet energy needs as well as reducing ammonia nitrogen emissions (Honorato et al., 2010; Kamalam et al., 2017). On the other hand, carbohydrates are also effective binding agents during the pelletization process (Arnesen and Krogdahl, 1993). As reported, some fish species, such as Atlantic salmon (*Salmo salar*) (Hemre and Hansen, 1998), grass carp (*Ctenopharyngodon idella*) (Gao et al., 2010), rainbow trout (*Oncorhynchus mykiss*) (Jin et al., 2014), and large yellow croaker (*Larimichthys crocea*) (Zhou et al., 2016), are capable of utilizing digestible carbohydrates to some extent. However, compared to mammals, high levels of carbohydrates in feed often lead to a reduction in fish growth and prolonged hyperglycemia, similar to type 2 diabetes (Wilson and Poe, 1987; Shi, 1997). The proportion of carbohydrates added to feed is usually low which has restricted the development of aquaculture. To address this problem, researchers have focused on the regulatory mechanism of glycometabolism in aquatic animals (Dalziel et al., 2018; Lu et al., 2018). Unfortunately, the regulatory mechanism of fish is not yet understood. Glucose is a polar molecule that relies on a transporter for transmembrane transport from the outer membrane to the inner membrane (Brant et al., 1993), and studies have shown that the family of specific facilitative glucose transporters (GLUTs) can mediate the absorption and distribution of glucose (Duehlmeier et al., 2007). The first rate-limiting step of these transporters is based on the diffusion gradient of glucose across cell membranes (Rose and Richer, 2005; Lu et al., 2018). Hence, the functional study of GLUTs continues to receive a great deal of attention (Barron et al., 2016).

In 1988, insulin-sensitive glucose transporter 4 (GLUT4) was identified for the first time in the study of insulin regulation of glucose metabolism (James et al., 1988). Thereafter, GLUT4 has been cloned in humans (Fukatmoto et al., 1989), rats (Charon et al., 1989), and mice (Kaestner et al., 1989). Initially, Wright et al. (1998) reported that GLUT4 was absent in teleosts fish, but this observation was due to lack of specific antibodies used for mammalian GLUT4 protein. Later, the presence of GLUT4 was demonstrated in brown trout (*Salmo trutta*) in 2000 (Planas et al., 2000), which proved that the presence of GLUT4 and paved the new way that glucose homeostasis is possibly regulated by the regulation of GLUT4 in fish. Specifically, it has been proven that GLUT4 is the most abundant glucose transporter in the skeletal muscle of mammals accounting for approximately 70% of the basal glucose transport, compared to the level reported in other tissues (Slot et al., 1991; Becker et al., 2001; Zorzano et al., 2005). In mammals, GLUT4 plays a central role in mediating insulin function to increase glucose uptake in the system of glucose metabolism homeostasis (Díaz et al., 2007; Marín-Juez et al., 2014). However, only a few studies have reported the role of GLUT4 in fish (Planas et al., 2000; Capilla et al., 2004a; Hall et al., 2006), besides, the mechanism is unclear and needs to be further investigated.

Blunt snout bream (*Megalobrama amblycephala*), a herbivorous freshwater fish widely found in China, Africa, North America, and Eurasia, has many advantages such as good meat quality and high economic value (Zhou et al., 2008). In China, the annual production output of this species reached nearly 800,000 tons in 2018 (Ministry of Agriculture, and Rural Affairs of the People’s Republic of China, Chinese Fisheries Yearbook, 2019). Like other fish species, blunt snout bream has limited ability to utilize carbohydrates, and adding high levels of carbohydrates in the feed may lower their growth performance and disturb glucose metabolism outcomes accompanied by persistent hyperglycemia (Ren et al., 2015). Unfortunately, to date, the reason for the carbohydrate intolerance in fish is not well understood. Moreover, information regarding the functional analysis and localization of GLUT4, as well as the mechanisms through which glucose levels are maintained in plasma remains unclear. Therefore, this study aimed to clone, identify the function of GLUT4, and to investigate its expression in response to carbohydrate fed to blunt snout bream.

## Materials and Methods

### Ethics Statement

The handling of the juveniles was in accordance with the Ministry of Agriculture, China, and international animal welfare laws, guidelines, and policies (Food and Agriculture Organization [FAO], 2004) and was approved by the Institutional Animal Care and Ethics Committee of Nanjing Agricultural University, Nanjing, China [Permit number: SYXK (Su) 2011-0036].

### Experimental Diets

Table 1 showed the various raw ingredients of feed used in the present study, and the diets were formulated to contain 30% glucose level. All the ingredients shown in Table 1 were ground into powder and sieved using a 40 mesh sieve. About 10% amount of water and 6% amount of oil were added to produce a stiff dough which was then forced through a granulator [F-26 (II), South China University of Technology, China] to make pellets. The pelleted diets were dried in a drying oven at 65°C. Finally, the pellets were stored at −20°C in sealed and well labeled plastic bags until required for use.

**TABLE 1:** Formulation and proximate composition of experimental diet for glucose feeding trial (% dry basis).

Ingredients			
Fish meal^1^	15	Cellulose^3^	11
Casein^1^	20	Carboxymethyl-cellulose	6
Gelatin^1^	5	Mineral premix^4^	1
Soybean oil	6	Vitamin premix^4^	1
Soybean lecithin	1	Monocalcium phosphate	2
Glucose^2^	30	Bentonite	2
**Composition analysis (Dry basis)**			
Crude protein	32.3		
Crude lipid	8.7		
Ash	9.8		
Gross energy (MJ/kg)	18.2		

*^1^Fish meal, obtained from Wuxi Tongwei feedstuffs Co., Ltd. (Wuxi, China), crude protein 65.4%, crude lipid 9.3%; Casein, obtained from Hua’an Biological Products Ltd. (Gansu, China), crude protein 90.2%; Gelatin, obtained from Zhanyun Chemical Ltd. (Shanghai, China), crude protein 90.1%. ^2^Glucose, obtained from Sigma-Aldrich. ^3^Microcrystalline cellulose, obtained from Xinwang Chemical Ltd. (Zhejiang, China). ^4^Mineral premix and vitamin premix were according to Ren et al. (2015).*

### Experimental Procedure

Healthy juveniles were selected from the Freshwater Fisheries Research Center (FFRC) of the Chinese Academy of Fishery Sciences breeding center. Before the experiment, the fish were acclimatized to experimental conditions in a water recirculatory system in cylindrical fiberglass tanks (300 L) for 15 days and fed with commercial diets containing 30.8% crude protein, 8.7% crude lipid, and 18.9% carbohydrate levels (Wuxi Tongwei feedstuffs Co., Ltd., China) to apparent satiation three times per day. At the end of the acclimatization period, 20 apparently healthy juvenile fish (initial weight 29.13 ± 0.21 g) were randomly distributed into every tank in triplicates, after which the feeding trial started, three times (08:00, 12:00, and 16:00) to apparent satiation based on the visual observation of fish feeding behavior. Throughout the experimental period, the water quality was tested once a week, the water temperature was maintained 28.13 ± 0.21°C, the pH at 7.22 ± 0.14, ammonia nitrogen values at 0.026 ± 0.002 mg/L, dissolved oxygen values at 6.43 ± 0.30 mg/L. A controlled photoperiod 12:12 h (light:dark) was maintained during the entire experiment.

### Sample Collection

After 63 days feeding trial, the fish (three fish per fiberglass tank) were randomly selected from each tank at different time points (1, 6, 12, and 24 h after feeding), and anesthetized using 100 mg/L MS-222. Immediately, blood samples were drawn from the caudal vein using disposable medical syringes a, and centrifuged at 3500 × *g* at 4°C for 10 min to allow for the collection of plasma. After this, the spleen, intestine, liver, muscle, gill, kidney, heart, and red blood cell samples were also collected into sterile tubes, and muscle were collected at 1, 6, 12, and 24 h after feeding. The collected samples were stored at −80°C in readiness for analysis.

### Cloning of GLUT4 cDNA

According to the previous description (Liang et al., 2018), RNAiso Plus reagent (TaKaRa, China) was used to extract the total RNA of mixed tissues. A reverse transcriptase M-MLV kit (TaKaRa, China) was used to synthesize the first-strand cDNA. Besides, 3′-full RACE Core Set Ver.2.0 kit and 5′-full RACE kit (TaKaRa, China) were used to perform 3′-rapid amplification of cDNA ends (RACE) and 5′-RACE. A gel extraction kit (Sangon, China) was used to purify the PCR products and sequenced on an ABI3730 DNA analyzer (ABI, United States) after insertion into a PMD-18T vector (TaKaRa, China). Table 2 showed all the primers used for cloning.

**TABLE 2:** Sequences of the PCR primers used in this work.

Use	Primer	Primer sequence (5′-3′)
CDS amplification	GLUT4-F	ATGCCGGCTGGGTTTCAGCAG
	GLUT4-R	TCAGTTCTCTGTCCCATCAG
3′RACE cloning	T3-1	TTTTGACCAGATCTCAGCCACCTTCCA
	T3-2	GCCTGGATCAAGGGAAACACAGTACAG
5′RACE cloning	T5-1	TCTTCTGTGGAGCGTT
	T5-2	GTAAGGAACCCAGTACAGCG
	T5-3	ACAGTGCGAGGGTTCCAG
Real-time primer	GLTU4-F	CCATTGCTGAGCTCTTTCGC
	GLUT4-R	GCGTACACTGGACTCTCCAC
	β-actin-F	TCGTCCACCGCAAATGCTTCTA
	β-actin-R	CCGTCACCTTCACCGTTCCAGT

*GLUT4: glucose transporter 4.*

### Plasma Parameters

Plasma total glucose (GLU) was measured using an automatic chemical analyzer, Mindray BS-400 (Shenzhen Mindray Biomedical Electronics Co., Ltd., China) following the description by Ren et al. (2017). In addition, an automatic chemiluminescence analyzer, Maglumi 1000, was used to determine plasma insulin levels (Snibe Diagnostic Ltd., China) according to the description of Liang et al. (2019).

### Immunohistochemical Analysis

In brief, muscle tissue was encased in resin through several processing steps, including paraformaldehyde fixation, alcohol dehydration, and paraffin embedding. Then, the normal paraffin sections were obtained using a LEICA slicer. Finally, immunohistochemical staining, including dewaxing and rehydration; antigen repair; primary antibody incubation; secondary antibody detection; alkaline phosphatase catalyzed color development; and other processes were performed.

### Quantitative Real-Time PCR Analysis of GLUT4 Expression

RNA was first extracted from samples using the RNAiso Plus kit (TaKaRa, China). The quality and quantity of RNA were assessed. After this, GLUT4 mRNA levels were determined using a 7500 real-time PCR machine (Applied Biosystems, United States). Initially, cDNA (2.0 μL) was reacted with 10.0 μL SYBR^®^ Premix Ex Taq II (2×), 0.8 μL forward and reverse primers (10 μM each), 0.4 μL ROX reference dye II (50×), and 6.0 μL RNase-free distilled water in a 20 μl final reaction volume. The specific primers of the target genes used were shown in Table 2. β-actin was used as a reference, non-regulated gene, and no obvious change was observed on its gene expression (Habte-Tsion et al., 2015). Relative genes expression was performed using Pfaffl’s mathematical model for CT calculation (Pfaffl, 2001).

### Western Blot Analysis

The method of western blot analysis was performed following the description by Liang et al. (2019). In brief, first, the lysate from the sample was prepared, and then western blot analysis was performed through the following main steps: (1) equal amounts of protein were loaded into the wells of the SDS-PAGE gel, (2) the proteins from the gel were transferred to a PVDF membrane, (3) the membrane was blocked using a blocking buffer, (4) the membrane was incubated with monoclonal antibody to GFP or GADPH diluted 1000× (Bio-Transduction Lab, China), (5) the membrane was washed, (6) the membrane was incubated with the of goat anti-mouse IgG-HRP diluted 1000× (Bio-Transduction Lab, China), (7) the secondary antibody was stained by a BeyoECL Star kit (Beyotime Biotechnology, China) based on the manufacturer’s instructions, and (8) the images were acquired using chemiluminescence. The polyclonal antibody against GLUT4 was synthesized based on the cloned GLUT4 sequence.

### Nucleotide Sequence and Bioinformatics and Statistical Analyses

The corresponding amino acid sequence was derived from the full-length cDNA sequence by NAman. The ProtParam tool was used to analyze the basic properties of the proteins^1^. PSORT Prediction software was used for the subcellular localization analysis^2^. For the domain analysis, NCBI conserved domains was used to identify the domain and functionality of the sequence (Marchler-Bauer et al., 2017)^3^. The SUMO and phosphorylation analyses were performed online using http://www.abcepta.com/sumoplot and http://www.cbs.dtu.dk/services/NetPhos/, respectively. The glycosylation analysis was performed via http://www.cbs.dtu.dk/services/NetNGlyc. Homologous sequence alignment was completed using ClustalX software, while the tertiary structure was predicted using the SWISS-MODEL (Biasini et al., 2014)^4^. A search for sequences similar to the GLUT4 amino acid sequence was carried out with the BLASTP program (Altschul et al., 1997) based on the NCBI website database, and the search results were verified with cluster analysis. MEGA7 software (Kumar et al., 2016) was used to perform multiple sequence alignment and build a phylogenetic tree (Saitou and Nei, 1987) with a bootstrap coefficient of 1000 based on the neighbor-joining (NJ) method. The evolutionary distance was calculated using the Poisson correction method (Zuckerkandl and Pauling, 1965).

The data collected from various parameters were subjected to a homogeneity test where necessary. Statistical analysis was performed using SPSS 16.0. Turkey’s test was used to show where the significant differences existed between the means. Data were expressed as means with SEM (M ± SEM).

## Results

### Cloning and Characterization of Blunt Snout Bream GLUT4 cDNA

**FIGURE 1:**
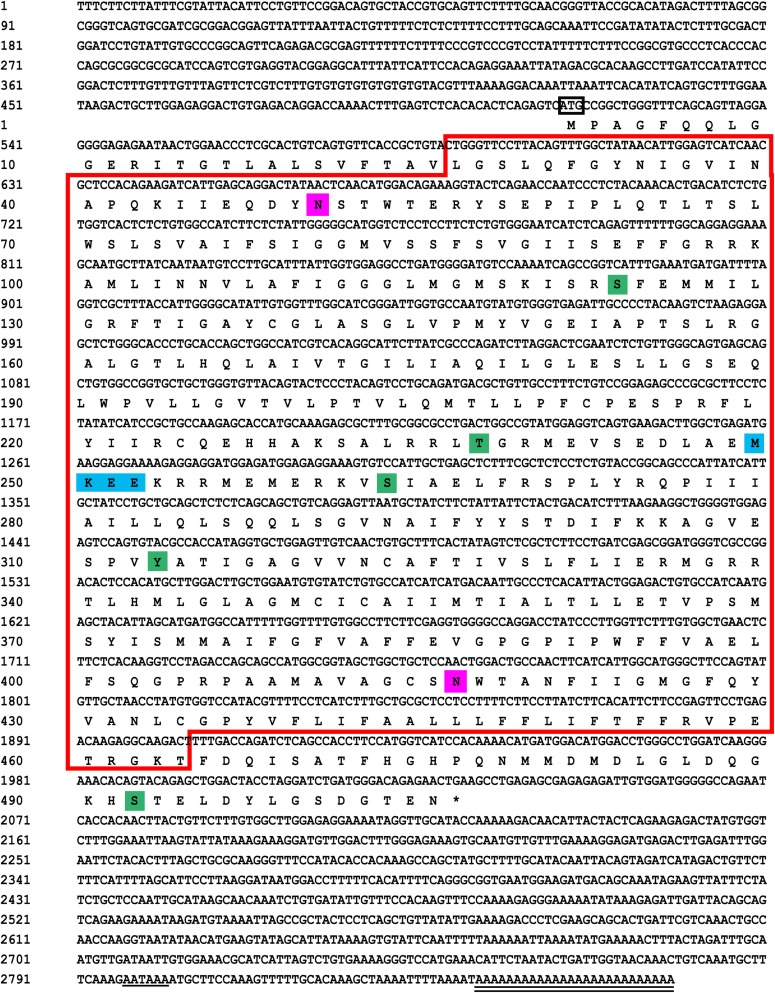
Sequencing results and amino acid sequence analysis of GLUT4 in *Megalobrama amblycephala*. The black box represents the ATG start codon; * represents the stop codon; underline represents the Poly (A) tail signal; double underline represents the poly (A) sequence; red box represents the possible Class 1 glucose transporters (GLUTs) of the Major Facilitator Superfamily structural domain (cd17431); purple shadow represents the possible N glycosylation sites; green shadow represents the possible phosphorylation site; blue shadow represents the possible SUMO site.

**FIGURE 2:**
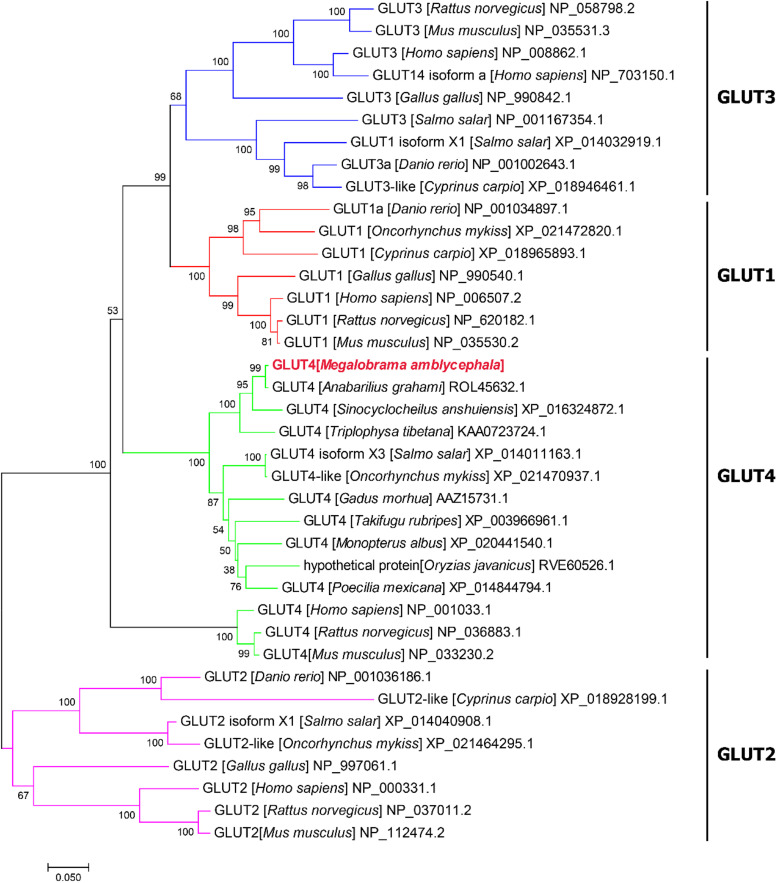
Phylogenetic tree of known vertebrate GLUT protein sequences. A phylogenetic tree was constructed with the complete protein sequence of blunt snout bream (*Megalobrama amblycephala*) GLUT4 (GenBank accession no: QLE11221), and a number of protein sequences corresponding to various vertebrate GLUTs.

**FIGURE 3:**
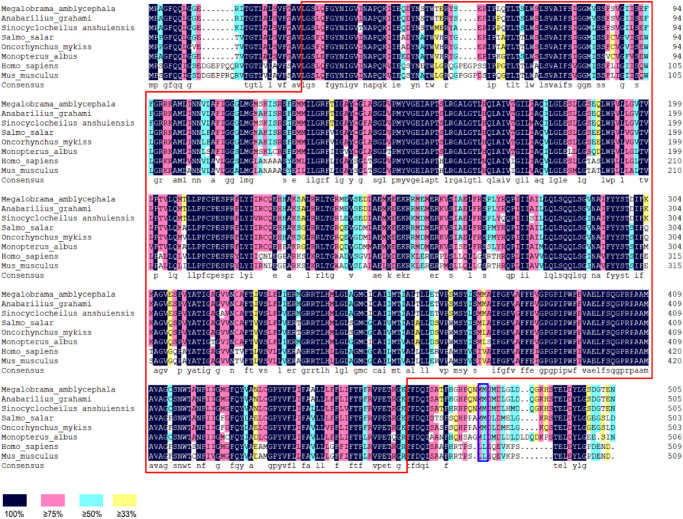
Homologous alignment of GLUT4 amino acid sequences in different species. Red box represents the Class 1 glucose transporters (GLUTs) of the Major Facilitator Superfamily structural domain (cd17431); blue box represents the dileucine internalization motif.

**FIGURE 4:**
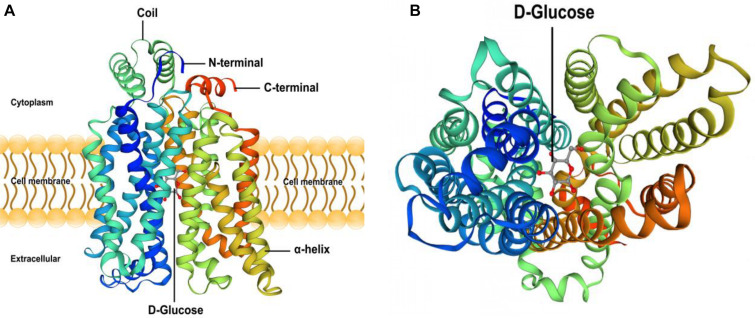
Protein 3D structure prediction of GLUT4 in *Megalobrama amblycephala*. Panel **(A)** shows that blue is the N-terminal, red is the C-terminal in **(A)**, GLUT4 was a transmembrane protein composed of a 12th-α-helix, with no β-pleated sheet, which formed a hollow channel and specifically identified monosaccharides transported across the membrane. Panel **(B)** shows that GLUT4 perspective to the bottom of the 3D conformation.

(1) The results showed that the GLUT4 cDNA sequence of blunt snout bream was 2868 bp long, and the mRNA contains a 5′-UTR region of 513 bp and a 3′-UTR region of 837 bp (GenBank accession no: MT447093). Through sequence alignment and bioinformatics analysis, it was inferred that the sequence was a full-length cDNA sequence (Figure 1). The GLUT4 cDNA sequence of the blunt snout bream has an open reading frame of 1518 bp and encodes 505 amino acids (Figure 1). GLUT4 had a theoretical isoelectric point and molecular weight were 6.41 and 55.47 kDa, respectively.
(2) Compared with the results of BLASTP analysis of the NCBI database, the Glut4 of blunt snout bream protein had the highest homology with Cypriniformes fish. The sequence similarity of GLUT4 in *Anabarilius grahami* (ROL45632.1), *Sinocyclocheilus anshuiensis* (XP_016324872.1), and *Triplophysa tibetana* (KAA0723724.1) was 99, 95, and 92%, respectively. Based on a phylogenetic tree generated from the GLUT1–4 amino acid sequences, glucose transporter cDNA from *M. amblycephala* was clustered with GLUT4 of Cypriniformes fish, and GLUT1, 2, 3, and 4 were clustered together (Figure 2).
(3) Based on the analysis of subcellular localization, GLUT4 was mainly found in the plasma membrane, accounting for 60.9% of the total expressed. Based on the results of GLUT4 similarity analysis, GLUT4 in aquatic animals contained multiple functional sites that regulated its intracellular transport, but it was also mutated. Compared with the data on *homo_sapiens* and *mus_musculus*, some of the mutations observed in the GLUT4 amino acid sequence of aquatic animals were FQQI mutated to FQQL, LL mutated to MM, and TELEY mutated to TELDY (Figure 3).
(4) Based on the results of the predicted tertiary structure, the possible transmembrane tertiary structure of GLUT4 was an outwardly closed conformation as shown in Figure 4A with blue for the N terminus and red for the C terminus and a transmembrane protein composed of a 12th α-helix with no β-pleated sheet, which formed a hollow channel and was specific for monosaccharides transported across the membrane (Figure 4B).

### Tissue Distribution of GLUT4

**FIGURE 5:**
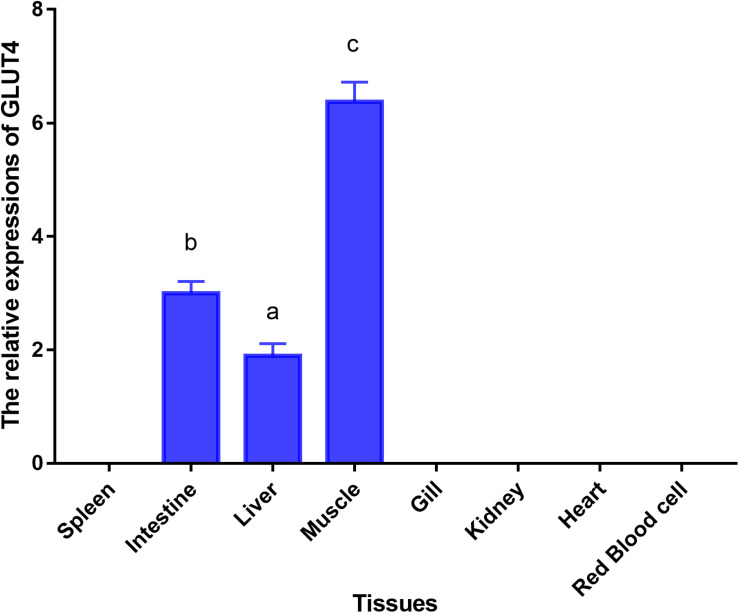
Tissue-specific mRNA expressions of GLUT4 in *Megalobrama amblycephala*. Vertical bars represent mean ± SE values for triplicate samples. Mean values with unlike small letters above bars are significantly different (*P* < 0.05).

**FIGURE 6:**
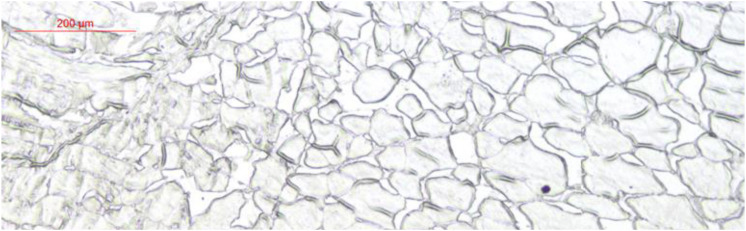
Immunohistochemical staining analysis in muscle of *Megalobrama amblycephala*.

Glucose transporter 4 mRNA was constitutively expressed in the muscle, intestine and liver. The highest mRNA level was observed in the muscle, and the mRNA levels of GLUT4 were not detected in the spleen, gill, kidney, heart, or red blood cells (Figure 5). Furthermore, immunohistochemical staining showed GLUT4 to be a kind of carrier protein embedded in the cell membrane to transport glucose and existed in the cell membrane (Figure 6).

### Continual Glucose Feeding Trial

**FIGURE 7:**
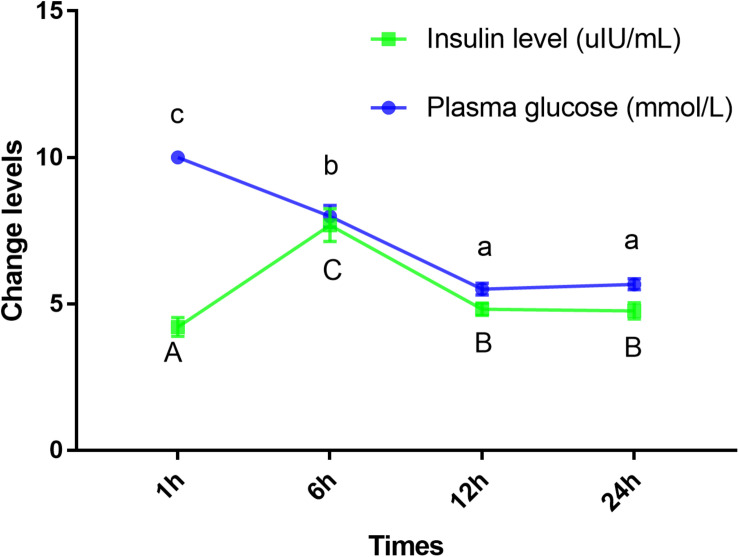
Insulin and glucose levels of plasma in *Megalobrama amblycephala*. Points represent mean ± SE values for triplicate samples. Mean values with unlike small letters or big letters in points are significantly different (*P* < 0.05).

**FIGURE 8:**
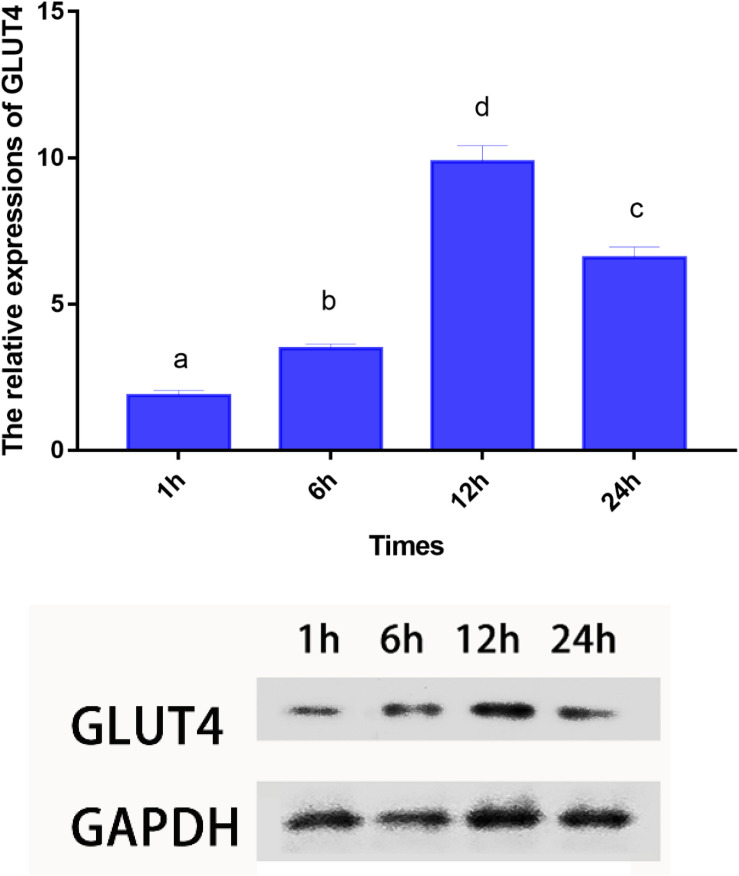
mRNA and protein expressions of GLUT4 in muscle of *Megalobrama amblycephala* after glucose intake. Vertical bars represent mean ± SE values for triplicate samples. Mean values with unlike small letters above bars are significantly different (*P* < 0.05).

Blood glucose level peaked within 1 h, however, the insulin levels peaked at 6 h. Furthermore, GLUT4 mRNA and protein levels of muscle showed an upward trend with an increase in feeding time, although, after 12 h, the expression and protein levels dropped sharply (Figures 7, 8).

## Discussion

Glucose transporter 4 has been identified in mammals (James et al., 1988) and some fish species (Capilla et al., 2004a; Li et al., 2018). In this study, the full-length cDNA of one glucose transporter from *M. amblycephala* (GenBank accession no: MT447093) was identified. Compared with the results of the BLASTP analysis using the NCBI database, the amino acid sequence of this gene showed the highest homology with Cypriniformes fish and glucose transporter cDNA from *M. amblycephala* clustered with GLUT4 of Cypriniformes fish based on a phylogenetic tree generated using GLUT1–4 amino acid sequences. Based on these results, the cDNA was categorized as GLUT4 of blunt snout bream (MaGLUT4). The results of the similarity analysis of GLUT4 among species showed that GLUT4 in aquatic animals contained multiple functional sites that regulated its intracellular transport, but it also had mutations. Compared with the *homo_sapiens* and *mus_musculus* sequence data, some mutations were observed in the GLUT4 amino acid sequence of aquatic animals including FQQI mutated to FQQL, LL mutated to MM and TELEY mutated to TELDY, indicating that the function and molecular characterization of GLUT4 probably further evolved depending on species differences. In humans, studies have revealed that the FQQI motif in the N terminus of GLUT4 plays an important regulatory role in the translocation of GLUT4 from the cell surface to the cell (Garippa et al., 1994), and TELEY, an acid cluster at one end, plays an important role in sorting and transporting GLUT4 within the cell under basic conditions (Blot and Mcgraw, 2008). Any mutation in either motif of GLUT4 alters the way GLUT4 is transported within the cell (Blot and Mcgraw, 2008). Additionally, it was noted that the LL functional sites, considered important regulators of GLUT4 translocation from intracellular vesicles to cell membranes and for endocytosis in mammals (Pessin et al., 1999; Al-Hasani et al., 2002; Capilla et al., 2007), were absent from the GLUT4 amino acid sequences in aquatic animals, which may be one of the reasons why fish are less tolerant to glucose than higher vertebrates.

In mammals, the skeletal, adipose tissue, and cardiac muscle are the main expression tissues of GLUT4, and are referred to insulin-sensitive tissues (Marín-Juez et al., 2014). In skeletal muscle, GLUT4, accounting for approximately 70% of the basal glucose transport of this tissue, is the major transporter expressed (Zorzano et al., 2005). In the present study, GLUT4 mRNA was constitutively expressed in the muscle, intestine, and liver, with the highest GLUT4 expression level observed in the muscle. Similar to the findings of the present study, the GLUT4 tissue expression pattern was also reported to be mainly distributed in the muscle tissue in mammals (Duehlmeier et al., 2007; Marín-Juez et al., 2014). In aquatic animals, the GLUT4 tissue expression pattern at the mRNA level has been examined in some species. The highest GLUT4 expression level was observed in the muscle in brown trout (Planas et al., 2000), tilapia (*Oreochromis niloticus* × *Oreochromis aureus*) (Chen, 2016), cobia (*Rachycentron canadum*) (Chen, 2016), pompano (*Trachinotus ovatus*) (Chen, 2016) and pearl gentian grouper (*Epinephelus fuscoguttatus* ♀ × *Epinephelus lanceolatus* ♂) (Li et al., 2018). However, in Atlantic cod (*Acipenser oxyrinchus*), GLUT4 was mainly distributed in the heart (Hall et al., 2006). The reason for the difference in the distribution of GLUT4 in different fish species remains unknown.

In this study, based on the analysis of sub-cellular localization, MaGLUT4 was mainly found in the plasma membrane, accounting for 60.9% of the total. Hence, MaGLUT4 might also be a membrane protein that acts as a transporter. Based on the immunohistochemical analysis, MaGLUT4 mainly existed in the muscle cell membrane. According to these results, MaGLUT4 was a kind of carrier protein embedded in the cell membrane to transport glucose, residing mainly in the cell membrane and playing a transmembrane transport role in blunt snout bream. The possible transmembrane tertiary structure of MaGLUT4 had an outwardly closed conformation, shown in Figure 4A as blue for the N-terminus and red for the C-terminus, with a transmembrane protein composed of a 12th α-helix with no β-pleated sheet, indicating that these structures formed a hollow channel that was specific for the monosaccharides transported across the membrane. The transition from an outwardly open to an outwardly closed conformation required a significant local rearrangement of the extracellular portion of the transmembrane segment, for which the C-terminal domain could provide the primary substrate-binding site. However, the possible tertiary transmembrane structure of GLUT4 in pompano was a β-pleated sheet (Chen, 2016). In the present study, the newly discovered MaGLUT4 possessed 12 transmembrane domains, one of the characteristics of GLUT4 in mammals (Joost and Thorens, 2001) and fish (Capilla et al., 2004a; Hall et al., 2006). Furthermore, this structure was in agreement with GLUTs reported in other fish, such as rainbow trout (Teerijoki et al., 2000), tilapia (*O. niloticus*) (Hrytsenko et al., 2010), gilthead seabream (*Sparus aurata*) (Balmaceda-Aguilera et al., 2012) and grouper (*Epinephelus coioides*) (Liu et al., 2017). These results indicated that GLUT4 amino acid sequences in fish were highly conserved compared to those in humans and mammals (Planas et al., 2000; Capilla et al., 2004a), a situation similar among other transporters. Overall, these results confirmed that MaGLUT4 was a structural and functional homolog of GLUT4 in mammals and other fish. However, the important difference was that GLUT4 in fish had a lower affinity for glucose and wider substrate specificity than mammalian GLUT4 (Díaz et al., 2007).

Mammals have a high capacity for glucose metabolism, and after consuming carbohydrates, blood glucose levels can return to normal within 1–2 h; however, the carbohydrate tolerance of fish is low and often accompanied by persistent hyperglycemia after carbohydrates are ingested (Moon, 2001). In this study, the plasma glucose levels peaked within approximately 1 h; furthermore, blood glucose levels returned to normal within 12 h, which indicated that blunt snout bream could digest glucose. These results were similar to those reported on other species, such as juvenile tilapia (Shiau and Chuang, 1995) and carp (*Cyprinus carpio*) (Stone, 2003). However, these results differed from those reported in carnivorous fish, such as white sturgeon (*Acipenser transmontanus*) (Hung, 1991), chinook salmon (*Oncorhynchus tshawytscha*) (Mazur et al., 1992), and rainbow trout (Polakof et al., 2012) where blood glucose peaked at least 6 h after carbohydrate intake with the recovery of basal blood glucose levels at least 18 h later. It was regrettable that the specific metabolic regulation mechanism was still unclear. Studies have shown that insulin is the most important regulator of blood glucose balance in mammals and fish (Viegas et al., 2013), and exogenous insulin could effectively reduce the blood glucose level of fish (Deroos et al., 1985). In the present study, the highest plasma insulin levels were observed within 6 h, which was consistent with previous results showing that ingesting digestible carbohydrates caused hyperglycemia and increased insulin levels (Capilla et al., 2004a, b). Interestingly, in this study, the blood glucose peaked within 1 h; while the highest level of insulin was observed after 6 h. These results indicated that fish might not respond to changes in plasma glucose in a timely manner. Therefore, the results of this study could be used to infer that this observation may be related to the delayed response mechanism of blood glucose changes, such as the delayed response of insulin to blood glucose changes, which might be an important cause of digestible carbohydrate intolerance in fish. This delay may be caused by the fish being stimulated to secrete glucagon and somatostatin hormones under the stress of hyperglycemia, thereby inhibiting the secretion of insulin and delaying the change in insulin in response to plasma glucose (Ronner and Scarpa, 1984; Harmon et al., 1991). Andoh (2015) also reported that the regulation of insulin levels in the plasma of barfin flounder (*Verasper moseri*), was mainly the result of release from Brockman bodies, not through transcription and translation, indicating that the mechanism of insulin release in teleosts might be different from that of glucose-dependent release in mammals (Hemre et al., 2002). As has been widely reported, insulin could activate GLUT4 (Lizunov et al., 2013), and the GLUT4 protein could be translocated from intracellular compartments to the plasma membrane to increase the uptake of glucose both in mammals (Bryant et al., 2002) and in fish (Lu et al., 2018). In this study, the mRNA and protein levels of GLUT4 showed an upward trend with an increase in feeding time; however, after 12 h, the expression level decreased sharply, indicating that GLUT4 was activated to maintain glucose homeostasis, thereby regulating and enhancing glucose metabolism in this fish. Similarly, dietary carbohydrate levels upregulated the expression of GLUT4 in giant grouper larvae (*E. lanceolatus*) (Lu et al., 2018), pearl gentian grouper (Li et al., 2018), and North Pacific spiny dogfish (*Squalus suckleyi*) (Deck et al., 2017) which contributes to the regulation of glucose metabolism. In fish, it has been proven that glucose can stimulate the secretion of insulin in a mammalian-like manner (Moon, 2001; Hrytsenko et al., 2008) which might account for the elevated mRNA and protein levels of GLUT4 in this study. Notably, some delays were observed in the changes of insulin and the expression of GLUT4 compared with the changes in plasma glucose. Similar to the results showing genetic enhancement in farmed tilapia, changes in insulin level and GLUT4 mRNA levels were delayed compared with the absorption rate of carbohydrates (Liu et al., 2018). Additionally, there was also a delayed response between changes in blood glucose and changes in insulin and mRNA levels of the genes in grouper (Mao, 2014). Overall, the results of this study indicated that plasma glucose adjustments caused by carbohydrate intake could be affected by the delayed action of insulin. This finding might explain why changes in the mRNA and protein levels of GLUT4 lagged notably behind the changes in blood glucose. Therefore, this delay in insulin level changes and GLUT4 activation might be an important reason for glucose intolerance in this fish.

## Conclusion

In general, we cloned the full length of MaGLUT4 and identified its functions and localization. MaGLUT4 is mainly distributed in the muscle and located in the cell membrane, where it played a role in transporting glucose into cells and regulating blood glucose. Furthermore, some delays were observed in the changes in insulin and the expression of GLUT4 compared with the changes in plasma glucose.

## Data Availability Statement

The raw data supporting the conclusions of this article will be made available by the authors, without undue reservation. Requests to access these datasets should be directed to MR.

## Ethics Statement

The handling of the juveniles was in accordance with the Ministry of Agriculture, China and international animal welfare laws, guidelines and policies (Food and Agriculture Organization [FAO], 2004) and was approved by the Institutional Animal Care and Ethics Committee of Nanjing Agricultural University, Nanjing, China [Permit number: SYXK (Su) 2011-0036].

## Author Contributions

XG and MR designed the study. HL carried out the experiments and wrote the manuscript. SM reviewed the manuscript. KJ provided technical assistance. HM provided technical guidance. All authors contributed to the article and approved the submitted version.

## Conflict of Interest

HM was employed by company Tongwei Co., Ltd. The remaining authors declare that the research was conducted in the absence of any commercial or financial relationships that could be construed as a potential conflict of interest.

## Footnotes

1 http://web.expasy.org/protparam

2 http://psort1.hgc.jp/form.html

3 https://www.ncbi.nlm.nih.gov/Structure/cdd/docs/cdd_search.html

4 https://swissmodel.expasy.org
